# An exploration of distinguishing subjective cognitive decline and mild cognitive impairment based on resting-state prefrontal functional connectivity assessed by functional near-infrared spectroscopy

**DOI:** 10.3389/fnagi.2024.1468246

**Published:** 2025-01-08

**Authors:** Zhengping Pu, Hongna Huang, Man Li, Hongyan Li, Xiaoyan Shen, Qingfeng Wu, Qin Ni, Yong Lin, Donghong Cui

**Affiliations:** ^1^Shanghai Key Laboratory of Psychotic Disorders, Shanghai Mental Health Center, Shanghai Jiao Tong University School of Medicine, Shanghai, China; ^2^Department of Psychogeriatrics, Kangci Hospital of Jiaxing, Tongxiang, Zhejiang, China

**Keywords:** subjective cognitive decline, mild cognitive impairment, functional near-infrared spectroscopy, machine learning, prefrontal cortex, resting-state functional connectivity

## Abstract

**Purpose:**

Functional near-infrared spectroscopy (fNIRS) has shown feasibility in evaluating cognitive function and brain functional connectivity (FC). Therefore, this fNIRS study aimed to develop a screening method for subjective cognitive decline (SCD) and mild cognitive impairment (MCI) based on resting-state prefrontal FC and neuropsychological tests via machine learning.

**Methods:**

Functional connectivity data measured by fNIRS were collected from 55 normal controls (NCs), 80 SCD individuals, and 111 MCI individuals. Differences in FC were analyzed among the groups. FC strength and neuropsychological test scores were extracted as features to build classification and predictive models through machine learning. Model performance was assessed based on accuracy, specificity, sensitivity, and area under the curve (AUC) with 95% confidence interval (CI) values.

**Results:**

Statistical analysis revealed a trend toward compensatory enhanced prefrontal FC in SCD and MCI individuals. The models showed a satisfactory ability to differentiate among the three groups, especially those employing linear discriminant analysis, logistic regression, and support vector machine. Accuracies of 94.9% for MCI vs. NC, 79.4% for MCI vs. SCD, and 77.0% for SCD vs. NC were achieved, and the highest AUC values were 97.5% (95% CI: 95.0%–100.0%) for MCI vs. NC, 83.7% (95% CI: 77.5%–89.8%) for MCI vs. SCD, and 80.6% (95% CI: 72.7%–88.4%) for SCD vs. NC.

**Conclusion:**

The developed screening method based on resting-state prefrontal FC measured by fNIRS and machine learning may help predict early-stage cognitive impairment.

## 1 Introduction

Neurocognitive disorders are a group of diseases characterized by varying degrees of cognitive impairment (Cheng et al., 2017). Dementia is the most advanced stage of cognitive impairment that causes immense suffering and imposes a huge burden on patients and their families (Mattap et al., 2022). Subjective cognitive decline (SCD) and mild cognitive impairment (MCI) are considered early-stage cognitive impairment and treated as potentially prodromal stages of dementia (Langa and Levine, 2014). Individuals with MCI and SCD typically present with the chief complaint of perceived decline in cognitive function. The main difference between these two conditions is that the impairments in objective cognitive examinations are only detected in MCI and not in SCD (Petersen et al., 2014; Rabin et al., 2017). Thus individuals with MCI have worse cognitive function than those with SCD. In previous studies, individuals with SCD or MCI were found to be at a much higher risk of progressing to dementia than normal controls (NCs) (Vos et al., 2015; Lee et al., 2020). However, it was estimated that timely intervention for early-stage cognitive impairment can delay the onset of dementia by 5 years, reducing the number of dementia cases by nearly 57% and saving almost half of related annual medical insurance costs (Brookmeyer et al., 2007). Thus, the development of accurate tools for the detection of SCD and MCI is imperative to help prevent the progression to dementia.

The existing methods to identify SCD or MCI have some shortcomings and limitations such as the subjectivity of neuropsychological tests, invasiveness of lumbar puncture, high cost of molecular imaging, ionizing radiation exposure with positron emission tomography (PET), confined space for magnetic resonance imaging (MRI), and the instability of electroencephalogram (EEG). Thus, there is a lack of simple screening tools with high specificity and sensitivity for the recognition of early-stage cognitive impairment. As a newly emerging optical imaging method, functional near-infrared spectroscopy (fNIRS) shows potential as an alternative modality due to its unique advantages (Nguyen et al., 2019; Yoo et al., 2020; Yu et al., 2020). It can help characterize brain activity by detecting the dynamic changes in oxyhemoglobin (HbO) and deoxyhemoglobin (HbR) according to the principle of neuro-vascular coupling (Ehlis et al., 2014; Huang et al., 2024). fNIRS provides higher spatial resolution than EEG, and higher temporal resolution than functional MRI (fMRI), with lower sensitivity to motion artifacts. It is a relatively less costly investigation that can be performed in individuals with metallic foreign bodies and those affected by claustrophobia. It also makes the experience better because of the low noise and lack of requirement for conductive paste. In addition, fNIRS does not entail exposure to ionizing injury. fNIRS has been used to explore the brain function in some neuropsychiatric diseases such as epilepsy (Rizki et al., 2015), schizophrenia (Okada et al., 2023), bipolar disorder (Aleksandrowicz et al., 2020), depression (Hu et al., 2021), anxiety (Zhang et al., 2023), autism (Lin et al., 2023), and sleep disorders (Mingming et al., 2024), and has shown satisfactory consistency with fMRI, PET, and EEG studies. However, there is a paucity of research on its applications in early-stage cognitive impairment (Li et al., 2018; Nguyen et al., 2019; Yoo et al., 2020; Yu et al., 2020; Kim et al., 2023).

The choice of indicator and the target encephalic region in fNIRS studies is another key issue. With an increasing number of studies on brain networks, atypical resting-state functional connectivity (FC) is increasingly regarded as one of the state markers in individuals with cognitive impairment (Li et al., 2016; Lin et al., 2018; Joshi et al., 2020; Soman et al., 2020). For example, patients with MCI were found to exhibit enhanced resting-state FC and over-activation of the brain default mode network (DMN), which was deemed as one of the compensatory mechanisms for initial cognitive impairment (Rashidi-Ranjbar et al., 2023). However, due to the antagonistic relationship between DMN and task-positive network (TPN), the compensatory activation of DMN will diminish the function of TPN, negatively affecting the cognitive function when performing tasks (Melrose et al., 2018). The prefrontal cortex (PFC) has extensive connections with other brain regions including the hippocampus, medial temporal lobe, and angular gyrus, which makes PFC an extremely important and complex region involved in the transmission and processing of information for mediating cognitive function (Miller and Cohen, 2001; Acunzo et al., 2022). Some parts of the PFC are the key components of DMN, which is now viewed as the core network impaired in Alzheimer’s disease (AD) (Jones et al., 2011). Some studies have found prefrontal dysfunction in individuals with early-stage cognitive impairment; however, these studies have yielded inconsistent or even contradictory results. For example, the FC between PFC and some brain regions was found to be weakened, while the prefrontal FC with other regions was strengthened (Nguyen et al., 2019; Li et al., 2020; Yu et al., 2020). Another limitation is the lack of focus on the FC between subregions within the PFC.

Consequently, we adopted the prefrontal resting-state PFC as the chief observation measure in this fNIRS study to unravel the characteristic changes in early-stage cognitive impairment. For more reliable results and to support the potential clinical diagnostic value of fNIRS in early-stage cognitive impairment, we employed machine learning to build models for screening MCI or SCD based on prefrontal resting-state FC and neuropsychological tests.

## 2 Materials and methods

### 2.1 Study design

This was a prospective, cross-sectional study enrolling individuals with SCD or MCI and matched healthy volunteers. All subjects received a series of neuropsychological tests covering the main cognitive domains and then underwent fNIRS scanning at resting state. The differences between these three groups were assessed. The second part of the study involved modeling to classify and predict early-stage cognitive impairment (SCD or MCI) based on near-infrared spectroscopic features of resting-state prefrontal FC and neuropsychological tests through machine learning.

A board-certified psychogeriatrist first interviewed the recruited participants and then a series of cognitive scales were used to screen participants for eligibility. The recruited participants were then divided into NC volunteers (NC group), SCD individuals (SCD group), and MCI individuals (MCI group) according to the inclusion and exclusion criteria. The common inclusion criteria of all enrolled participants were: (1) male or female subjects aged ≥55 years; and (2) Mini-Mental State Examination (MMSE) score >19 (for those with primary school education) or >24 (for those with high school education or more) (Pu et al., 2020). Participants in the MCI group were also required to qualify the following criteria: (1) International Working Group diagnostic criteria for MCI (Winblad et al., 2004); and (2) clinical dementia rating (CDR) score ≤0.5 (Pu et al., 2020), Montreal Cognitive Assessment (MoCA) score ranging from 18 (inclusive) to 26 (Nasreddine et al., 2005; Roalf et al., 2013), and Functional Activities Questionnaire (FAQ) score ≥9 (González et al., 2022). Participants in the SCD group were required to qualify the following criteria: (1) the diagnostic criteria of the SCD Initiative (SCD-I) Working Group (Jessen et al., 2014); and (2) CDR score = 0 (Pu et al., 2020), MoCA score ≥26 (Nasreddine et al., 2005; Roalf et al., 2013), Subjective Cognitive Decline Questionnaire 9 (SCD-Q9) score ≥5 (Hao et al., 2017), and FAQ score <9 (González et al., 2022). The inclusion criteria for the NC group were as follows: (1) no subjective feeling of cognitive decline; (2) normal results of objective cognitive tests; and (3) CDR score = 0 (Pu et al., 2020), MoCA score ≥26 (Nasreddine et al., 2005; Roalf et al., 2013), SCD-Q9 score <5 (Hao et al., 2017), and FAQ score <9 (González et al., 2022). The common exclusion criteria were: (1) severe cognitive impairment such as dementia or intellectual disability; (2) serious neuropsychiatric disorders affecting cognitive function; (3) use of medication or other therapeutic methods that may affect cognitive function; (4) somatopathy that affects cognitive function; (5) somatic conditions affecting cerebral oxygen supply; and (6) inability to understand the instructions of the neuropsychological tests. Finally, 246 participants including 55 NCs, 80 SCD individuals, and 111 MCI individuals were enrolled.

Written informed consent was obtained from each participant or his/her legal guardian at the time of enrollment. This study was approved by the ethics committees of the Kangci Hospital of Jiaxing and Shanghai Mental Health Center. This study is registered with the Chinese Clinical Trial Registry (registry number: ChiCTR2300067594).

### 2.2 Neuropsychological tests

#### 2.2.1 General cognitive function

The MMSE was adopted to assess the general cognitive function. The scale consists of 30 questions with one point awarded for each correct answer. This examination covers six domains, i.e., orientation, immediate memory, attention and calculation, short-term memory, language, and visuospatial skills. The cutoff scores are set according to different educational levels. A score ≤19 in individuals with primary school education or a score ≤24 in individuals with high school education or beyond is suggestive of dementia (Pu et al., 2020).

#### 2.2.2 Attention and processing speed

Part A of the Trail-making Test (TMT-A) was chosen to appraise the attention and processing speed. In this test, the participants are asked to draw a line to connect 25 consecutive numbers (from 1 to 25) which are randomly distributed on an A4-sized page. The time taken to complete this test (measured in seconds) is recorded as the score. A score of ≥72.5 s is considered indicative of possible impairment in attention and processing speed (Wei et al., 2018).

#### 2.2.3 Executive function

The Chinese version of part B of the Trail-making Test (TMT-B) was used to assess executive function. In this test, the participants are asked to draw a line to alternately connect 25 numbers enclosed in circles or squares (——…—) which are randomly distributed on an A4-size page. The time spent in accomplishing this test (measured in seconds) is recorded as the score. A score of ≥135.5 s is considered indicative of possible impairment in executive function (Wei et al., 2018).

#### 2.2.4 Language function

The Chinese version of the Boston Naming Test (BNT) was employed to evaluate the language function, which has been shown to have satisfactory validity for detecting naming skills in Chinese-speaking populations. This test is composed of 30 pictures presented as line patterns, and the participants are required to name each picture within 20 s. One point is awarded for each correct answer. A score of ≤22 indicates possible impairment in language function (Li et al., 2022).

#### 2.2.5 Memory

The Hopkins Verbal Learning Test (HVLT) consists of examinations of short-term memory, delayed memory, and recognition which may take at least 30 min. For the purpose of this study and to improve the compliance of participants, we only assessed short-term memory. The examiner first read 12 nouns aloud, with a 2-s time interval between each word. The participants were then asked to recall all these nouns immediately with no limitation of sequence. This procedure was repeated three times, and one point was awarded for each correct recall. A score of ≤21.5 is considered indicative of possible impairment in short-term memory (Shi et al., 2012).

#### 2.2.6 Visuospatial skill

The Clock-Drawing Test (CDT) was used to assess visuospatial skills. The subjects were required to draw a clock on a blank A4 paper according to this two-step instruction: first, draw a 10-cm diameter clock face with all numbers (1–12) on it; second, place the hour hand and minute hand in the correct positions to make the clock show 11:10. The CDT was scored following the criterion of Cahn et al. (1996) (10-point rating scale). The total score is 10 points: clock face (0–2 points), placement of the hands (0–4 points); and placement of the numbers (0–4 points). Higher scores indicate better visuospatial skills. A CDT score (criterion of Cahn et al., 1996) ≤7 is considered indicative of impairment in visuospatial skills (Yamamoto et al., 2004).

### 2.3 fNIRS measurement and data reprocessing

**FIGURE 1:**
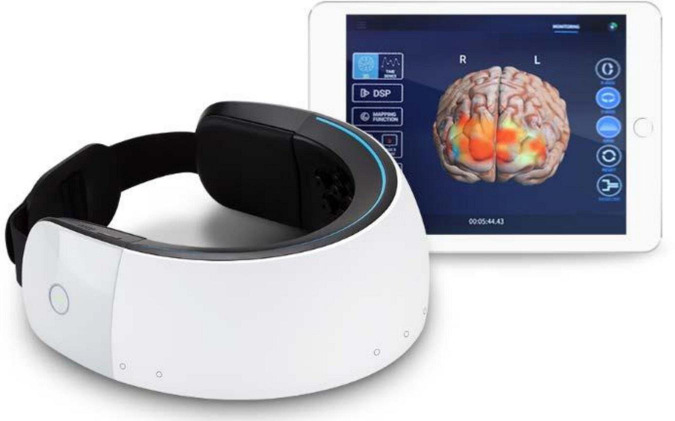
NIRSIT apparatus and tablet computer (cited from https://www.obelab.com/product/product_nirsit.php).

A portable and multichannel fNIRS device named NIRSIT (OBELAB, Seoul, Republic of Korea) was used to measure the dynamic changes in HbO and HbR at wavelengths of 780 and 850 nm to reveal the strength of prefrontal FC (Figure 1). The primary parameters of NIRSIT including the sampling rate, spatial resolution, and time resolution were 8.138 Hz, 4 mm × 4 mm, and 125 ms/8 Hz, respectively. There were 24 sources and 32 detectors in this device. The source and detector distance of NIRSIT was 3.0 cm which was considered the most representative observational depth in the PFC and can optimally avoid the interference of blood flow in the scalp and bone, corresponding to a total of 48 channels in PFC (Funane et al., 2014; Brigadoi and Cooper, 2015; Yoo et al., 2020). The NIRSIT was connected to a tablet computer (Galaxy Tab, Samsung, Republic of Korea) through WLAN communication, and the fNIRS data were recorded by a built-in program and software in the tablet computer.

The fNIRS measurement was performed in a confined and quiet room after the neuropsychological tests, with no interference from other strong artificial light sources and electromagnetic signals. The participants sat still in a comfortable chair with ease. Sweat and oil secreted by the skin were first cleaned using medical alcohol swabs before the placement of NIRSIT probes over the forehead. Due care was taken to ensure that the probes were not blocked or interfered with by hair on the forehead and temples. Then the built-in program of gain calibration was automatically conducted to obtain the optimal signals, and the program of eliminating motion artifacts was initiated to improve the quality of measurement. Finally, all participants underwent 5 min fNIRS measurement at resting state.

The Homer2 was employed to reprocess the fNIRS data. Channels with the signal-to-noise power ratio <25 dB were excluded as the bad channels. Then the near-infrared signals were transformed into optical density using the “hmrIntensity2OD” function. The functions of “hmrMotionArtifactByChannel” (tMotion = 2, tMask = 4, STDEVthresh = 50, AMPthresh = 5) and “hmrMotionCorrectSpline” (*P* = 0.99, turnon = 1) were applied to remove and correct artifacts (Guerrero-Mosquera et al., 2016; Nguyen et al., 2019). Subsequently, the optical density data were bandpass filtered to eliminate the instrument and global physiological noise using the “hmrBandpassFilt” function (hpf = 0.01, lpf = 0.1) (Nguyen et al., 2019; Yu et al., 2020; Zhang et al., 2022). To ensure data quality, the program of principal component analysis (PCA), which is built into the software of Homer2, was performed to further eliminate the artifacts and superficial global physiological noise (Wilcox et al., 2005; Brigadoi et al., 2014). Finally, the optical density data were transformed into dynamic changes of HbO, HbR, and total hemoglobin (HbT) using the “hmrOD2Conc” function (ppf = 6) (Hiraoka et al., 1993; Herold et al., 2018).

### 2.4 Prefrontal FC

**FIGURE 2:**
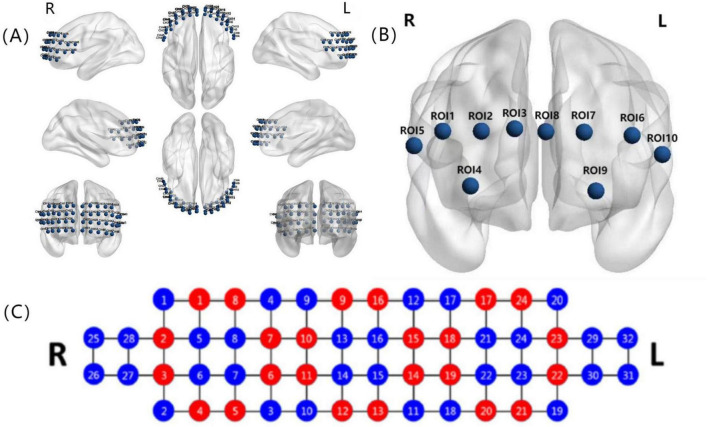
**(A)** The three-dimensional view of 48 prefrontal channels; **(B)** the arrangement of 10 ROIs; and **(C)** the distribution of 24 sources (red) and 32 detectors (blue) in NIRSIT. ROI 1 included channels 1, 2, 6, 7, 11, and 12; ROI 2 included channels 34, 35, 37, 38, 42, and 43; ROI 3 contained channels 3, 8, 13, 17, 21, and 25; ROI 4 contained channels 20, 24, 28, 33, 36, and 41; ROI 5 covered channels 18, 22, and 26; ROI 6 covered channels 19, 23, and 27; ROI 7 involved channels 14, 15, 16, 29, and 30; ROI 8 involved channels 31, 32, 46, 47, and 48; ROI 9 included channels 4, 5, 9, and 10; and ROI 10 covered channels 39, 40, 44, and 45. ROI, region of interest.

The 48 prefrontal fNIRS channels were divided into 10 regions of interest (ROIs) according to the anatomical automatic labeling (AAL) provided by the Montreal Neurological Institute (MNI), and the locations of functional subregions in the PFC via NIRS-SPM (Ye et al., 2009; Szczepanski and Knight, 2014; Akamatsu et al., 2016), including the right dorsolateral prefrontal cortex (DLPFC) (ROI 1), left DLPFC (ROI 2), right DLPFC (majority) and right rostral prefrontal cortex (RPFC) (minority) (ROI 3), left DLPFC (majority) and left RPFC (minority) (ROI 4), right medial prefrontal cortex (MPFC) (ROI 5), left MPFC (ROI 6), right orbitofrontal cortex (OFC) (ROI 7), left OFC (ROI 8), right ventrolateral prefrontal cortex (VLPFC) (ROI 9), and left VLPFC (ROI 10) (Figure 2).

In this study, the relative concentrations of HBO, HBR, and HBT for all channels in each ROI were averaged to obtain time series for HBO, HBR, and HBT. Then the methods of amplitude-frequency-based coherence (COH) (Wang et al., 2014) and phase locking value (PLV) (Zhang et al., 2016) were used to calculate the FC between pairs of ROIs within the PFC, both with a value range of 0–1. A value closer to 1 indicates a stronger correlation or phase synchronization.

### 2.5 Classification and prediction models

Classification and prediction models were built through a machine learning approach and based on the resting-state FC under the time series for HbO, HbR, and HbT signals along with the scores of MMSE, TMT-A, TMT-B, BNT, HVLT, and CDT, which generated 270 FC connections (2 × 3 × 10 × 9/2) and 6 neuropsychological features. To avoid overfitting and underfitting during the training phase, and to ensure the generalizability of models across different fold numbers and subject-specific variations, 5-fold and 10-fold cross-validation were adopted successively to train and test the classifier (Ding et al., 2022; Kaliappan et al., 2023; Hakimi et al., 2024). We first used *z*-score normalization for the training sets (Zhang et al., 2022); then, features with variance ≤1 were removed and PCA dimensionality reduction was applied to filter redundant and repetitive features (Ringnér, 2008; Ding et al., 2022). Finally, the performance of the models was assessed by calculating recognition accuracy (ACC), specificity (SPE), sensitivity (SEN), and area under the curve (AUC) with 95% confidence interval (CI) in the test set (Ding et al., 2022).

The linear discriminant analysis (LDA), logistic regression (LR), Gaussian Naive Bayes (GNB), support vector machine (SVM), *k*-nearest neighbor (KNN), random forest (RF), extreme gradient boosting (XGBoost), gradient boosting decision tree (GBDT), and random undersampling boosting (RUSboost) were adopted as the models for classification and prediction. The classification and prediction tasks involved SCD vs. NC, MCI vs. NC, and MCI vs. SCD.

### 2.6 Statistical analysis

Differences among the three groups regarding demographic features, neuropsychological scales, and FC strengths were first evaluated by analysis of variance (ANOVA) using MATLAB 2021a if the data were normally distributed or approximately normally distributed. Non-normally distributed data were transformed to a normal distribution by *z*-score normalization. The false discovery rate (FDR) correction was then employed to rectify *P* values (Singh and Dan, 2006; Glickman et al., 2014). Lastly, the post-hoc pairwise comparison was performed by applying the least-significant difference *t* (LSD-*t*) test if the corrected ANOVA *P* < 0.05. *P* < 0.05 indicated a statistical difference, while *P* < 0.01 was considered indicative of a significant statistical difference.

As to the model construction, the permutation test was performed to determine the empirical chance level accuracy (EACC) (Ding et al., 2022). ACC > EACC means that the performance of a given model is better than those built from randomly shuffled datasets under equal conditions (Ding et al., 2022). AUC ≥0.79 indicates better performance of the models (de Hond et al., 2022).

## 3 Results

### 3.1 Demographic and cognitive features at baseline

Fifty-five NC volunteers (NC group), 80 SCD patients (SCD group), and 111 MCI patients (MCI group) were included in this study. There were no significant between-group differences with respect to age or sex (*P* > 0.05). The scores of MMSE, TMT-A, TMT-B, BNT, HVLT, and CDT in the MCI group were significantly lower than those in the NC and SCD groups (*F*_(2,243)_ = 76.52, *P* < 0.001; *F*_(2,243)_ = 66.28, *P* < 0.001; *F*_(2,243)_ = 74.03, *P* < 0.001; *F*_(2,243)_ = 95.55, *P* < 0.001; *F*_(2,243)_ = 189.57, *P* < 0.001; *F*_(2,243)_ = 69.63, *P* < 0.001, respectively). The general demographic information of the three groups as well as the results of neuropsychological tests are summarized in Table 1.

**TABLE 1:** General demographic data and results from neuropsychological tests.

Item	Group			*F*/χ ^2^	*P*
	**NC**	**SCD**	**MCI**		
Age (years)	61.44 ± 4.46	63.30 ± 6.54	64.80 ± 6.60	0.55	0.578
Sex (male/female)	24/31	41/39	58/53	1.17	0.557
MMSE score	28.91 ± 1.20	28.35 ± 1.30	25.49 ± 2.56	76.52	<0.001
TMT-A score (s)	40.73 ± 12.47	57.41 ± 19.85	98.22 ± 45.44	66.28	<0.001
TMT-B score (s)	59.51 ± 22.59	86.45 ± 28.51	186.31 ± 102.84	74.03	<0.001
BNT score	27.29 ± 1.65	25.41 ± 2.67	20.63 ± 4.04	95.55	<0.001
HVLT score	26.91 ± 3.27	21.85 ± 4.23	13.50 ± 5.13	189.57	<0.001
CDT score	9.33 ± 0.71	8.73 ± 1.10	7.50 ± 1.63	69.63	<0.001

MMSE, Mini-Mental State Examination; TMT-A, Trail-making Test A; TMT-B, Trail-making Test B; BNT, Boston Naming Test; HVLT, Hopkins Verbal Learning Test; CDT, Clock-Drawing Test; NC, normal control; SCD, subjective cognitive decline; MCI, mild cognitive impairment.

### 3.2 Resting-state FC results based on ROI

Among the 246 study participants, one did not complete the fNIRS study due to an unbearable feeling of fullness in the head, and 42 others were excluded from the final analysis due to the low quality of their data. Consequently, 203 participants, including 48 NC volunteers (NC group), 65 SCD patients (SCD group), and 90 MCI patients (MCI group) were included in the FC analysis. The following results represented the FC within the PFC as derived from three types of hemodynamic responses (HbO, HbR, and HbT) at the resting state.

**FIGURE 3:**
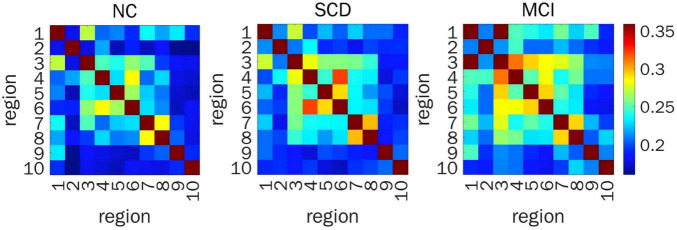
Resting-state prefrontal FC from the time series of HBR calculated by COH. Heat map of FC matrix in the three groups, where warmer color indicates stronger FC. FC, functional connectivity; HBR, deoxyhemoglobin; ROIs, regions of interest; COH, coherence; FDR, false discovery rate; NC, normal control; SCD, subjective cognitive decline; MCI, mild cognitive impairment; ROI 1, right DLPFC; ROI 2, left DLPFC; ROI 3, right DLPFC (majority) and right RPFC (minority); ROI 4, left DLPFC (majority) and left RPFC (minority); ROI 5, right MPFC; ROI 6, left MPFC; ROI 7, right OFC; ROI 8, left OFC; ROI 9, right VLPFC; ROI 10, left VLPFC.

When representing FC by COH, significant differences in prefrontal FC were detected between ROI 1 and ROI 3, and ROI 2 and ROI 7 (*F*_(2,200)_ = 7.44, *P* = 0.029; *F*_(2,200)_ = 6.88, *P* = 0.029, respectively) from the time series for HbR. *Post hoc* pairwise comparisons showed that the FC between ROI 1 and ROI 3, and between ROI 2 and ROI 7 in MCI individuals was stronger than that in NC volunteers (LSD-*t* = 2.76, *P* = 0.006; LSD-*t* = 2.94, *P* = 0.004, respectively) and SCD individuals (LSD-*t* = 2.85, *P* = 0.005; LSD-*t* = 2.58, *P* = 0.011, respectively) (Figure 3).

**FIGURE 4:**
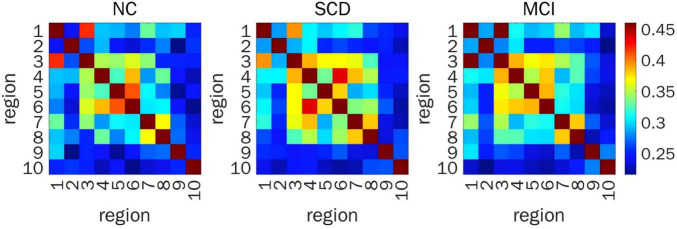
Resting-state prefrontal FC from the time series of HBR calculated by PLV. Heat map of FC matrix in the three groups, where warmer color indicates stronger FC. FC, functional connectivity; HBR, deoxyhemoglobin; PLV, phase locking value; ROIs, regions of interest; FDR, false discovery rate; NC, normal control; SCD, subjective cognitive decline; MCI, mild cognitive impairment; ROI 1, right DLPFC; ROI 2, left DLPFC; ROI 3, right DLPFC (majority) and right RPFC (minority); ROI 4, left DLPFC (majority) and left RPFC (minority); ROI 5, right MPFC; ROI 6, left MPFC; ROI 7, right OFC; ROI 8, left OFC; ROI 9, right VLPFC; ROI 10, left VLPFC.

When representing FC by PLV, a significant difference in prefrontal FC was detected between ROI 4 and ROI 7 (*F*_(2,200)_ = 11.02, *P* = 0.001) from the time series for HbR. *Post hoc* pairwise comparisons showed that the FC between ROI 4 and ROI 7 in SCD individuals was stronger than that in NC volunteers (LSD-*t* = 3.61, *P* < 0.001) and SCD individuals (LSD-*t* = 3.91, *P* < 0.001) (Figure 4).

Irrespective of COH or PLV, there was no statistical difference between the three groups in prefrontal FC from the time series for HbO and HbT (*P* > 0.05).

### 3.3 Performance of machine learning-based models

#### 3.3.1 Models only based on resting-state FC

For distinguishing SCD from NC, the ACC and AUC values for all nine models were unsatisfactory (<65.0%). The GBDT showed the best and most balanced performance, with an ACC of 68.1%, an AUC of 62.6%, an SPE of 31.3%, and an SEN of 87.8%.

For distinguishing MCI from NC, the models with the best and most balanced performance were the LDA (ACC: 68.8%; AUC: 70.8%; SPE: 43.8%; and SEN: 82.2%) and LR (ACC: 69.6%; AUC: 70.0%; SPE: 43.8%; and SEN: 83.3%).

**FIGURE 5:**
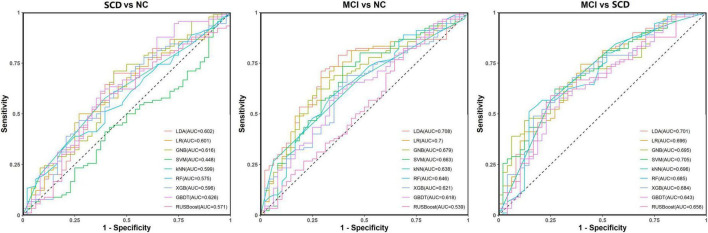
Receiver operating characteristic (ROC) curves for the abilities of the nine models to complete classification tasks based on resting-state prefrontal functional connectivity alone.

For distinguishing MCI from SCD, the model with the best and most balanced performance was the SVM (ACC: 71.0%; AUC: 70.5%; SPE: 41.7%; and SEN: 86.7%). The results for the abilities of the nine models to perform classification and prediction tasks based only on resting-state FC are presented in Figure 5 and Table 2.

**TABLE 2:** Performance metrics for nine models for classification tasks based on resting-state prefrontal FC alone.

Task	Model	ACC	EACC	SPC	SEN	AUC	95% CI of AUC
SCD (positive class) vs. NC (negative class)	LDA	0.652	0.638	0.375	0.800	0.602	0.506–0.699
	LR	0.623	0.638	0.333	0.778	0.601	0.505–0.698
	GNB	0.638	0.638	0.313	0.811	0.616	0.521–0.713
	SVM	0.616	0.638	0.354	0.756	0.448	0.347–0.551
	KNN	0.616	0.638	0.188	0.844	0.599	0.502–0.696
	RF	0.630	0.667	0.063	0.933	0.575	0.477–0.674
	XGBoost	0.652	0.645	0.354	0.811	0.596	0.497–0.692
	GBDT	0.681	0.652	0.313	0.878	0.626	0.532–0.721
	RUSBoost	0.580	0.601	0.542	0.600	0.571	0.471–0.669
MCI (positive class) vs. NC (negative class)	LDA	0.688	0.638	0.438	0.822	0.708	0.621–0.794
	LR	0.696	0.645	0.438	0.833	0.700	0.614–0.788
	GNB	0.652	0.638	0.250	0.867	0.679	0.589–0.769
	SVM	0.674	0.638	0.375	0.833	0.663	0.572–0.755
	KNN	0.638	0.645	0.146	0.900	0.638	0.544–0.732
	RF	0.645	0.667	0.104	0.933	0.646	0.552–0.739
	XGBoost	0.630	0.645	0.292	0.811	0.621	0.526–0.716
	GBDT	0.645	0.652	0.250	0.856	0.618	0.521–0.713
	RUSBoost	0.601	0.601	0.354	0.733	0.539	0.439–0.639
MCI (positive class) vs. SCD (negative class)	LDA	0.681	0.638	0.375	0.844	0.701	0.615–0.789
	LR	0.674	0.638	0.396	0.822	0.696	0.610–0.784
	GNB	0.674	0.638	0.229	0.911	0.695	0.608–0.784
	SVM	0.710	0.638	0.417	0.867	0.705	0.619–0.793
	KNN	0.674	0.645	0.146	0.956	0.696	0.609–0.784
	RF	0.696	0.667	0.208	0.956	0.685	0.597–0.775
	XGBoost	0.652	0.638	0.292	0.844	0.684	0.596–0.774
	GBDT	0.652	0.652	0.271	0.856	0.643	0.550–0.737
	RUSBoost	0.630	0.601	0.396	0.756	0.656	0.563–0.747

#### 3.3.2 Models based on FC and neuropsychological tests

For distinguishing SCD from NC, the LR model had the best ACC (77.0%) and the LDA had the largest AUC (80.6%). The models with the most balanced performance were the LDA (ACC: 75.2%; AUC: 80.6%; SPE: 66.7%; and SEN: 81.5%), LR (ACC: 77.0%; AUC: 79.1%; SPE: 64.6%; and SEN: 86.2%), and SVM (ACC: 75.2%; AUC: 74.6%; SPE: 62.5%; and SEN: 84.6%).

For distinguishing MCI from NC, the LR had the best ACC (94.9%) and the LDA had the largest AUC (97.5%). The models with the most balanced performance were the LDA (ACC: 92.0%, AUC: 97.5%, SPE: 91.7%, and SEN: 92.2%), LR (ACC: 94.9%, AUC: 97.2%, SPE: 87.5%, and SEN: 98.9%), and SVM (ACC: 90.6%, AUC: 97.2%, SPE: 87.5%, and SEN: 92.2%).

**FIGURE 6:**
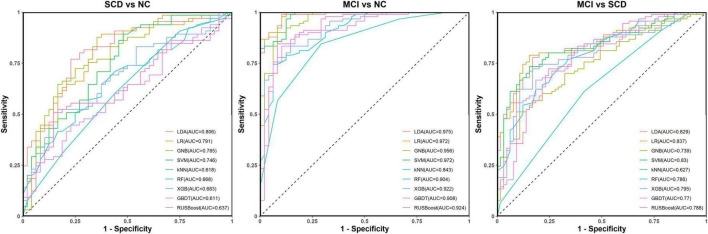
Receiver operating characteristic (ROC) curves for the abilities of the nine models to complete classification tasks based on resting-state prefrontal functional connectivity and neuropsychological tests.

For distinguishing MCI from SCD, the LDA showed the best ACC (79.4%) and the LR had the largest AUC (83.7%). The models showing the most balanced performance were the LDA (ACC: 79.4%, AUC: 82.9%, SPE: 78.5%, and SEN: 80.0%) and LR (ACC: 77.4%, AUC: 83.7%, SPE: 73.8%, and SEN: 80.0%). The performance results of the nine models for classification and prediction tasks based on both FC and neuropsychological tests are shown in Figure 6 and Table 3.

**TABLE 3:** Performance metrics for nine models for classification tasks based on resting-state prefrontal FC and neuropsychological tests.

Task	Model	ACC	EACC	SPC	SEN	AUC	95% CI of AUC
SCD (positive class) vs. NC (negative class)	LDA	0.752	0.592	0.667	0.815	0.806	0.727–0.884
	LR	0.770	0.592	0.646	0.862	0.791	0.710–0.872
	GNB	0.717	0.592	0.667	0.754	0.785	0.702–0.867
	SVM	0.752	0.592	0.625	0.846	0.746	0.657–0.835
	KNN	0.584	0.592	0.563	0.600	0.618	0.515–0.721
	RF	0.611	0.602	0.375	0.785	0.668	0.569–0.767
	XGBoost	0.619	0.592	0.458	0.738	0.683	0.587–0.781
	GBDT	0.566	0.592	0.333	0.738	0.611	0.507–0.714
	RUSBoost	0.575	0.584	0.479	0.646	0.637	0.535–0.738
MCI (positive class) vs. NC (negative class)	LDA	0.920	0.638	0.917	0.922	0.975	0.950–1.000
	LR	0.949	0.638	0.875	0.989	0.972	0.944–1.000
	GNB	0.891	0.638	0.875	0.900	0.956	0.926–0.986
	SVM	0.906	0.638	0.875	0.922	0.972	0.943–1.000
	KNN	0.797	0.652	0.708	0.844	0.843	0.780–0.906
	RF	0.833	0.667	0.563	0.978	0.904	0.855–0.952
	XGBoost	0.819	0.645	0.729	0.867	0.922	0.880–0.964
	GBDT	0.826	0.652	0.667	0.911	0.908	0.860–0.955
	RUSBoost	0.862	0.601	0.833	0.878	0.924	0.882–0.966
MCI (positive class) vs. SCD (negative class)	LDA	0.794	0.587	0.785	0.800	0.829	0.766–0.891
	LR	0.774	0.587	0.738	0.800	0.837	0.775–0.898
	GNB	0.684	0.587	0.662	0.700	0.739	0.662–0.816
	SVM	0.742	0.587	0.646	0.811	0.830	0.767–0.892
	KNN	0.600	0.587	0.585	0.611	0.627	0.539–0.714
	RF	0.697	0.600	0.431	0.889	0.786	0.716–0.856
	XGBoost	0.729	0.587	0.646	0.789	0.795	0.726–0.863
	GBDT	0.697	0.587	0.585	0.778	0.770	0.697–0.842
	RUSBoost	0.729	0.574	0.646	0.789	0.788	0.718–0.858

## 4 Discussion

Our results revealed the characteristic differences of resting-state prefrontal FC between NC and individuals with SCD and MCI. First, the resting-state prefrontal FCs in early-state cognitive impairment were stronger than those in NCs. Secondly, individuals with early-stage cognitive impairment exhibited more atypical cross-hemisphere FCs than those confined within the ipsilateral cerebral hemisphere. Thirdly, the most atypical regions of the prefrontal FC in individuals with early-stage cognitive impairment were the DLPFC (ROIs 1, 2, 3, and 4) and the OFC (ROI 7). Finally, all atypical prefrontal FCs were found under the time series for HbR.

To develop an optimal classification and predication model for early-stage cognitive impairment, we employed nine widely used models including LDA, LR, GNB, SVM, KNN, RF, XGBoost, GBDT, and RUSboost to perform the classification and prediction tasks. We also took the general trend into account rather than only features showing statistical differences between groups. We built models from two methods, one was based on resting-state FC alone, while the other was based on the combination of FC and neuropsychological tests. The models only based on FC showed unsatisfactory performance in classification tasks, especially for the task of SCD vs. NC. Both ACC and AUC were close to 70.0% when performing tasks of MCI vs. NC and MCI vs. SCD. These indices were much improved when a combination of FC and neuropsychological tests was used. The best accuracies of 94.9% for MCI vs. NC, 79.4% for MCI vs. SCD, and 77.0% for SCD vs. NC were achieved, and the highest AUC values were 97.5% (95% CI: 95.0%–100.0%) for MCI vs. NC, 83.7% (95% CI: 77.5%–89.8%) for MCI vs. SCD, and 80.6% (95% CI: 72.7%–88.4%) for SCD vs. NC.

As the key subregions involved in cognitive function, the DLPFC and OFC are the important components of resting-state intrinsic connected networks (Kazemi et al., 2018; Jin et al., 2019), which have close connections with many other cerebral areas, such as the parietal cortex, intraparietal sulci, and sensory-motor cortex (Zhou et al., 2020; Jung et al., 2022). Such connections provide anatomic and histological foundations for these prefrontal subregions to mediate cognitive function including working memory, learning, planning, attention, motive, behavioral inhibition, behavioral decision-making, emotion, and social control (Miller and Cohen, 2001).

The human brain is increasingly considered as a network (Yu et al., 2020). The connected mode of each brain region or subregion is just like its unique fingerprint to distinguish itself from others, which also endows specific functions (Luo et al., 2024). Thus, FC analysis is an effective method to explore cognitive function. Previous studies have demonstrated atypical resting-state FC in both early-stage cognitive impairment and dementia (Teipel et al., 2018; Zhang et al., 2022). Thus, as the core cerebral region associated with cognitive function, the exploration of prefrontal FC at the resting state is of great significance. Previous fMRI and EEG studies have shown atypical FCs between the MPFC and hippocampus, the left OFC and left dorsal preinsular lobe, the PFC and parietal lobe, and the PFC and posterior areas in individuals with SCD (Lazarou et al., 2020; Viviano and Damoiseaux, 2021; Ulbl and Rakusa, 2023). There were more atypical prefrontal FCs in MCI individuals, such as between the right OFC and other regions including left superior temporal gyrus, precentral gyrus, right thalamus, left fusiform gyrus, right precuneus, and right cuneus; between the DLPFC and other regions including the hippocampus, left posterior cingulate, and bilateral anterior insula; and between subregions within PFC (Zhen et al., 2018; Min et al., 2019; Liang et al., 2020; Chen et al., 2022). These resting-state FCs in SCD and MCI individuals are usually enhanced to compensate for cognitive impairment (Behfar et al., 2020).

Only a few studies have investigated resting-state FC using fNIRS in SCD individuals. However, these studies showed over-activated PFC in SCD, which might indicate some compensatory mechanism in SCD individuals (Teo et al., 2021; Salzman et al., 2023; Lee et al., 2024). There were relatively more studies investigating resting-state FC using fNIRS in MCI individuals and these studies have not only found atypical long-range connections of PFC with the parietal lobe, occipital lobe, and right temporal lobe (Yeung and Chan, 2020), but also revealed over-activation in FC between the subregions within PFC (Nguyen et al., 2019; Behfar et al., 2020; Liang et al., 2020). Effective connectivity was also found to be significantly lower between the bilateral PFC (Bu et al., 2019). Notably, recent fNIRS studies suggested the potential of this technology to identify MCI based on atypical FCs between the right DLPFC and left parietal lobe, with an ACC of 73.86% (Zhang et al., 2022). However, there is a paucity of fNIRS studies focusing on changes in FC among prefrontal subregions. Our results are consistent with those of previous studies and also display atypical FCs among prefrontal subregions.

As a rapidly advancing automatic learning method, machine learning can help identify complex non-linear correlations in high-latitude data, and this approach can facilitate early identification of cognitive impairment (Merkin et al., 2022). Several recent studies have employed machine learning to build models for the classification and prediction of various kinds of cognitive impairment based on neuroimaging data. Two recent resting-state EEG studies reported ACC values ranging from 63.95% to 93.88% for the classification task of MCI vs. NC by machine learning (Ding et al., 2022; Perez-Valero et al., 2022). According to a meta-analysis, the ACC of resting-state fMRI through machine learning ranged from 62.00% to 93.29% for MCI vs. NC (Ibrahim et al., 2021). A recent resting-state fNIRS study using machine learning reported ACC values ranging from 71.59% to 73.86% for MCI vs. NC (Zhang et al., 2022). Recent studies using neuropsychological tests as features to distinguish MCI reported ACC values ranging from 66.22% to 91.30% and AUC values ranging from 65.00% to 95.00% (Dong et al., 2022; Kurbalija et al., 2023; Gómez-Valadés et al., 2024). A recent meta-analysis revealed that the ACC and AUC of fluid biomarkers to identify MCI ranged from 61.00% to 97.00%, and 75.00% to 97.00%, respectively (Blanco et al., 2023). Similar to our study, the ACC and AUC values in the above-cited studies were lower when only based on one kind of feature such as EEG, fMRI, fNIRS, neuropsychological tests, and fluid biomarkers alone. However, the ACC and AUC values became much higher when integrating multiple-modality features. In addition, the above studies shared some common limitations. For example, the classification of individuals with SCD was overlooked, and most of the studies did not include neuropsychological test results as extracted features that play a much more fundamental role than other methods in screening cognitive impairment. Another important flaw was that most of these studies only extracted features with obvious statistical differences, but ignored general trends. This approach may result in overfitting and an artificially inflated ACC. In contrast, the classification tasks in our study included SCD individuals and we employed neuropsychological test scores covering major cognitive domains as extracted features. Moreover, we took the general trends of FC into account to reduce the possibility of overfitting.

Some limitations of this study should be considered and addressed in future research. The foremost limitation is the cross-sectional nature of the study. A longitudinal study for predicting the progression of SCD or MCI to dementia is required to prove the clinical value of fNIRS. The process of participant selection is another problem. SCD and MCI can result from multiple etiologies including AD-related and non-AD-related causes. Despite the relatively strict inclusion and exclusion criteria in this study, we did not assess etiological factors. A longitudinal study with a 3- to 5-year follow-up based on the present study and more etiological investigations such as apolipoprotein E genotyping, ^18^F-florbetaben PET amyloid or tau protein imaging, or lumbar puncture should be performed to obtain more robust evidence. A combination of other objective parameters and more advanced deep learning methods can be introduced to build more optimal models.

## 5 Conclusion

The present study demonstrated the potential value of fNIRS for discriminating SCD and MCI based on resting-state prefrontal FC and neuropsychological tests. To build upon these results, longitudinal studies with larger sample sizes, etiological examinations, more comprehensive parameters, and more advancing learning methods are warranted.

## Data Availability

The raw data supporting the conclusions of this article will be made available by the authors, without undue reservation.
